# Sensitive electrochemical detection of p-nitrophenol by pre-activated glassy carbon electrode integrated with silica nanochannel array film

**DOI:** 10.3389/fchem.2022.954748

**Published:** 2022-08-05

**Authors:** Ruobing Su, Hongliang Tang, Fengna Xi

**Affiliations:** ^1^ Department of Chemistry, Key Laboratory of Surface & Interface Science of Polymer Materials of Zhejiang Province, Zhejiang Sci-Tech University, Hangzhou, China; ^2^ Affiliated Fangchenggang Hospital, Guangxi University of Chinese Medicine, Fangchenggang, China

**Keywords:** electrochemical sensor, nanochannel array film, pre-activated glassy carbon electrode, sensitive detection, p-nitrophen

## Abstract

Convenient, rapid and sensitive detection of p-nitrophenol (p-NP), one of the priority environmental pollutants, in environmental samples is of great significance. Electrochemical sensor with simple fabrication process, high sensitivity and selectivity, good antifouling, and regeneration performance is highly desirable. Herein, an electrochemical sensing platform is demonstrated based on the integration of vertically-ordered mesoporous silica-nanochannel film (VMSF) on electrochemical pre-activated glassy carbon electrode (p-GCE), which is able to realize ultrasensitive detection of p-NP in environmental samples. Electrochemical pre-activation of GCE is achieved through a simple and green electrochemical polarization process including anodic oxidation at high voltage and the following cathodic reduction at low voltage. The p-GCE possesses enhanced active area and introduced active sites, and enables stable binding of VMSF. VMSF is easily grown on p-GCE through the electrochemically assisted self-assembly (EASA) method within 10 s. Owing to the hydrogen bonding between silanol groups and p-NP, VMSF nanochannels display strong enrichment effect for the detection of p-NP. The developed VMSF/p-GCE sensor can achieve sensitive detection of p-NP ranging from 10 nM to 1 μM and from 1 to 30 μM with a limit of detection (LOD) of 9.4 nM. Considering the antifouling ability of VMSF, detection of p-NP in pond water is achieved.

## 1 Introduction

Environmental pollution, especially environmental water pollution, has received more and more attention due to its great harm to human health and ecological environment (Cheng et al., 2021; Li et al., 2022). Nitrophenols are an important class of environmental pollutants with high chemical stability, low biodegradability and severe toxicity. As an aromatic nitrophenol compound, p-nitrophenol (p-NP) is widely used as an intermediate of fine chemicals such as pesticides, medicines, and dyes (Yuan et al., 2019; Chakraborty et al., 2021; Zhao et al., 2021). For instance, it can be used to prepare various sulfurized dyes or dye intermediate 4-aminophenol. In the pharmaceutical industry, p-NP is applied to synthesize phenacetin and paracetamol. In addition to being discharged through industrial wastewater, p-NP can also be released into environment through the widespread use of organophosphorus pesticides in agriculture since it is the hydrolysed product of some organophosphorus pesticides (e.g., nitroparathion, methyl parathion and ethyl parathion) (Dzyadevych and Chovelon, 2002). However, p-NP is highly toxic and carcinogenic and can persist in the environment for a long time. Generally, short-term exposure to p-NP causes headaches, nausea, and drowsiness, as well as irreversible damage to human liver and kidney (Mulchandani et al., 2005; Wang et al., 2015; Chakraborty et al., 2021). The US Environmental Protection Agency (EPA) regulates p-NP as one of the priority environmental pollutants (Niazi and Yazdanipour, 2007; Qiu et al., 2007; Menazea and Mostafa, 2020). Therefore, convenient, rapid and sensitive detection of p-NP in environmental samples is of great significance.

At present, the methods for p-NP detection include spectrophotometry, capillary electrophoresis, high performance liquid chromatography, Raman spectroscopy, etc. (Niazi and Yazdanipour, 2007; Qiu et al., 2007; Danhel et al., 2009; Iqbal, 2011; Vijayarangamuthu and Rath, 2014; Menazea and Mostafa, 2020) However, these strategies often suffer from the problems of complex detection process, high cost, and slow speed. Electrochemical methods have the advantages of rapid detection, low cost, simple instrumentation, easy integration, and suitability for miniaturization. With the development of nanotechnology, the sensitivity of electrochemical detection can be further improved by modifying electrodes with appropriate nanomaterials. For instance, p-NP could be sensitively detected based on electrodes modified with molecularly imprinted polyaniline on graphene oxide flakes (Saadati et al., 2018), cyclodextrin-modified gold nanoparticles (CD-AuNPs) on mesoporous carbon (MC) (Zhou et al., 2019), or polyvinylpyrrolidone (PVP) modified black phosphorus (BP) nanocomposite (Shen et al., 2021). However, contamination and interference on electrodes in analysis of complex samples can significantly reduce the accuracy and stability of electrochemical (bio)sensors. This is due to the fact that complex samples tend to have complex matrices. On the one hand, particles or coexisting macromolecules in complex matrices tend to contaminate electrodes. On the other hand, electrochemically active molecules coexisting in the sample also interfere with the assay. Thus, tedious pre-treatment processes such as separation is commonly needed in electrochemical detection of complex samples. Overcoming contamination and interference issues and enabling direct electrochemical detection (electroanalysis without tedious pre-treatment such as separation) of complex samples remains a challenge. Facile fabrication of electrochemical sensor through equipment appropriate nanomaterials with common electrode to improve the anti-interference and antifouling performance and detection sensitivity is highly desirable.

Vertically-ordered mesoporous silica-nanochannel film (VMSF) has a highly ordered nanochannel array, which is perpendicular to the electrode surface and has high pore density (∼40,000 μm^−2^) as well as uniform diameter (mesopore size, typically 2–3 nm) (Lin et al., 2016; Sun et al., 2016; Walcarius, 2021). These unique structure characteristics endow VMSF with excellent permeability and ensure rapid diffuse of small molecules to the surface of electrode. It has been proved that the detection sensitivity can be significantly improved by modifying the electrode with VMSF. This is attributed to the significant enrichment of VMSF towards target analytes resulting from the high specific surface area of VMSF and multi-interactions (e.g., electrostatic interactions, hydrogen bonding, etc. (Yan et al., 2020; Yan et al., 2021; Ma et al., 2022a; Wang et al., 2022a)) with the analytes. As known, the silica structure of VMSF contains a large number of silanol groups (p*K*_a_ 2–3), so that the nanochannels exhibit remarkable charge selectivity because they tend to carry a net negative charge after dissociation of silanol groups. For example, Cheng et al. reported that VSMF can electrostatically enrich Pb^2+^ to remarkably improve the detection sensitivity (Cheng et al., 2018). Su et al. found that VSMF can electrostatically enriched electrochemiluminescence probes (tris(2,2-bipyridyl) dichlororuthenium (II), Ru (bpy)_3_^2+^) with positive charge, leading to the improvement of the detection sensitivity by 2 orders of magnitude (Zhou et al., 2015; Ma et al., 2022b). Our group reported the confinement of zero-dimensional (0D) graphene quantum dots in nanochannels to further improve the enrichment effect as well as the detection sensitivity (Lu et al., 2018). In contrast to these electrostatic enrichment, small redox species with the same charges to nanochannels hardly reach the electrode surface due to the charge selective permeability, leading to anti-interference ability of VMSF-modified electrode (Zhou et al., 2018). In addition, ultrasmall nanochannels also show remarkable size selectivity. Proteins, cells, starch and other large particles or macromolecules are excluded from VMSF nanochannels (Serrano et al., 2015; Vilà et al., 2016; Liu et al., 2020; Liang et al., 2021; Zhou et al., 2022). Therefore, VMSF-modified electrodes have great potential in direct detection of p-NP in environmental samples owing to excellent antifouling and anti-interference properties.

In this paper, we established an electroanalysis platform for rapid, highly sensitive, and direct detection of p-nitrophenol (p-NP) in environment samples by integrating VMSF on an electrochemically pre-activated glassy carbon electrode (p-GCE). Electrochemical polarization including anodic oxidation at high potential and the following cathodic reduction at low potential is applied as the simple and green method to pre-activate GCE. The resulting p-GCE exhibits high electroactive area and high electrocatalytic performance. VMSF can be stably grown on p-GCE surface by electrochemical self-assembly method (EASA) (Walcarius et al., 2007). Based on the enrichment by nanochannels, VMSF/p-GCE enables highly sensitive detection of p-NP. Combined with the antifouling properties of VMSF, the developed VMSF/p-GCE sensor can be used for direct electrochemical detection of p-NP in environmental water. The constructed electrochemical sensor has remarkable advantages of simple construction method, convenient operation, low electrode preparation cost and high detection sensitivity.

## 2 Materials and methods

### 2.1 Chemicals and materials

Tetraethyl orthosilicate (TEOS), cetyltrimethylammonium bromide (CTAB), potassium ferricyanide (K_3_ [Fe(CN)_6_]), potassium ferrocyanide (K_4_ [Fe(CN)_6_]), catechol (CC), sodium dodecyl sulfate (SDS), starch and humic acid (HA), p-nitrophenol (p-NP) were purchased from Shanghai Aladdin Biochemical Technology Co., Ltd. Hydroquinone (HQ) and p-aminophenol (p-AP) were obtained from Shanghai Macklin Biochemical Technology Co., Ltd. Sodium nitrate (NaNO_3_) was provided from Wuxi Zhanwang Chemical Reagent Co., Ltd. Bovine serum albumin (BSA) was purchased from sigma Aldrich. Ethanol (99.8%), sodium chloride (NaCl), calcium chloride (CaCl_2_), potassium chloride (KCl) and ferric chloride (FeCl_2_) were obtained from Hangzhou Gaojing Fine Chemical Co., Ltd. Concentrated hydrochloric acid (HCl) was purchased from Hangzhou Shuanglin Chemical Reagent Co., Ltd. Phosphate buffer was prepared using Na_2_HPO_4_ and NaH_2_PO_4_. The pond water was taken from a pond of Zhejiang Sci-Tech University (Hangzhou, China). All chemicals and reagents were used directly without further purification. The aqueous solution in the experiment was prepared with ultrapure water (18.2 MΩ cm). Glassy carbon electrodes (GCE, 3 mm in diameter) were purchased from Shanghai CHI Instrument Co., Ltd. Before use, GCE was polished with 0.5, 0.3 and 0.05 μm alumina slurries on a polishing cloth, respectively. The obtained electrode was then ultrasonically cleaned in ethanol and ultrapure water for 1 min, respectively.

### 2.2 Measurements and instrumentations

The morphology of VMSF was characterized by transmission electron microscopy (TEM). TEM measurements were carried out with an HT7700 transmission electron microscopy (TEM, Japan) at an accelerating voltage of 100 kV. The VMSF samples were prepared by gently scraping VMSF from the surface of the p-GCE. After dispersed in ethanol under ultrasound, the sample was dropped onto the supporting copper mesh. Electrochemical impedance spectroscopy (EIS), cyclic voltammetry (CV), and differential pulse voltammetry (DPV) were carried out at room temperature on an Autolab (PGSTAT302N) electrochemical workstation (Metrohm, Switzerland). A conventional three-electrode system was adopted, with Ag/AgCl as the reference electrode, platinum electrode as the counter electrode, and bare GCE or modified GCE as the working electrode. For DPV measurement, the step potential was 0.005 V with pulse amplitude of 0.05 V and pulse time of 0.05 s. The interval time was 0.2 s.

### 2.3 Preparation of vertically-ordered mesoporous silica-nanochannel film on electrochemical activated glassy carbon electrode

Clean GCEs were firstly pre-activated through electrochemical polarization before VMSF growth. Electrochemical polarization consists of two steps of anodization and cathodic reduction. Briefly, anodization was performed by applying a constant voltage of +1.8 V at GCE for 300 s in PBS (0.1 M, pH 5.0). The followed cathodic reduction was carried out by cyclic voltammetry with a potential scan range of −1.3 to 1.25 V for three consecutive cycles. After being thoroughly rinsed with ultrapure water, the electrochemically pretreated electrode was obtained and named as p-GCE.

Vertically-ordered mesoporous silica-nanochannel film (VMSF) was grown on p-GCE by electrochemically assisted self-assembly (EASA) method. The precursor solution for VMSF growth was prepared by adding tetraethyl orthosilicate (TEOS, 2.833 g) and alkyltrimethylammonium bromide (CTAB, 1.585 g) in the mixture of ethanol (20 ml) and NaNO_3_ solution (20 ml, 0.1 M, pH 2.6) followed with stirring for 2.5 h. To grow VMSF, p-GCE was immersed in the precursor solution and a constant current density (−0.74 mA/cm^2^) was applied for 10 s. Subsequently, the electrode was thoroughly washed with ultrapure water. Then, Scotch tape was used to clean the surface several times to remove possible agglomerates of silica. The obtained VMSF was aged at 80°C overnight. The resulting electrode contained a large amount of surfactant micelles that filled in the nanochannels and denoted as SM@VMSF/p-GCE. The inner micelle templates could be removed by immersing the SM@VMSF/p-GCE in HCl solution (0.1 M in ethanol) and stirring for 5 min. The obtained modified electrode with open nanochannels was named VMSF/p-GCE.

### 2.4 Electrochemical detection of p-nitrophenol

For electrochemical detection of p-NP, PBS (0.1 M, pH 3.0) was selected as the supporting electrolyte. Under the optimal conditions, different concentrations of p-NP were added in the electrolyte. After enrichment by stirring for 75 s, DPV curves were recorded. In the real sample analysis, the pond water was filtered using a nylon filter (0.22 μm) and then diluted by a factor of ten. Then, p-NP was electrochemically detected using standard addition method. For real sample analysis, the workshop wastewater containing p-NP was provided by Hangzhou agrochemical Co., Ltd. (Hangzhou, China). The wastewater was analyzed after diluted using the electrolyte. For comparison, the p-NP was also detected using high performance liquid chromatography (HPLC, Agilent 1,260 Series) equipped with ZORBAX SB-C18 column (5 μm, 4.6 × 250 mm) and UV detector at 318 nm (mobile phase: methanol/water 1:1; flowing rate: 1.0 ml/min, column temperature: 55°C, quantifying method: external standard method, WS/T 58–1996).

## 3 Results and discussion

### 3.1 Convenient integration of vertically-ordered mesoporous silica-nanochannel film on electrochemically pre-activated glassy carbon electrode

**FIGURE 1:**
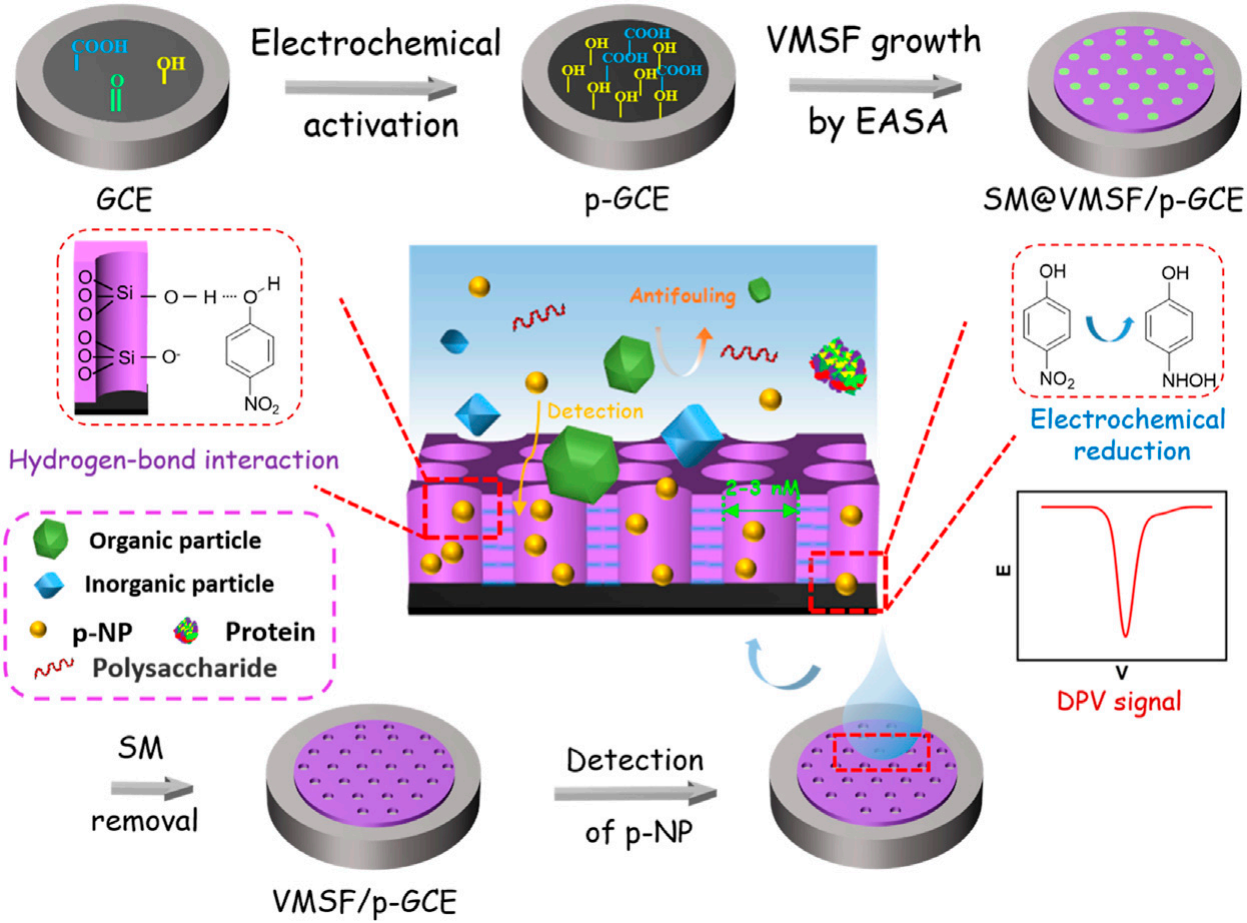
Schematic illustration for the fabrication of electrochemical sensor based on equipment of VMSF on electrochemical pre-activated GCE and the following detection of p-NP.

Figure 1 illustrates the facile fabrication of the sensors and subsequent electrochemical detection of p-NP based on the integration of VMSF on electrochemically pre-activated GCE (p-GCE). GCEs are the most commonly used electrochemical electrodes. When VMSF is directly grown on the surface of GCE, however, the VMSF film cannot exist stably and can be peeled off by rinsing with ultrapure water. Stable binding of VMSF on GCE, carbon fiber, and Au electrode has been pioneered using organosilane (e.g., electrografting of 3-aminopropyltriethoxysilane) as the molecular glue (Nasir et al., 2018; Gong et al., 2021). On the other hand, electrochemical pre-activation process also provides a convenient method to stably grow VMSF on GCE (Li et al., 2019). It has been proven that electrochemical pre-activation can increase the active surface area of the electrode and introduce oxygen-containing functional groups on electrode surface, which will act as active sites to enhance the adsorption of analyte (e.g., through electrostatic adsorption or hydrogen bonding, etc.) and interfacial electron transfer. Thus, electrochemical pre-activation of GCE is firstly performed on GCE, which is realized using a simple and green electrochemical polarization method. The pre-activation of GCE involves two steps of an anodization and a followed cathodic reduction. The former can generate oxygen-containing groups such as carboxyl, carbonyl, and hydroxyl groups on the surface of electrode, but it reduces the conductivity of the electrode. The latter restores the conductivity of the electrode while reducing part of the carbonyl groups to hydroxyl groups. The change of surface chemistry for GCE caused by electrochemical pre-activation was characterized by X-ray photoelectron spectroscopy (XPS). Although the high-resolution C1s spectra of both bare GCE and p-GCE reveal four types of carbon bonds including C–C/C=C (sp (Li et al., 2022) carbon, 284.4 eV), C–O (285.8 eV), C=O (287.1 eV), and O–C=O (288.6 eV) (Supplementary Figure S1), the C–O content significantly increased in p-GCE, indicating the generation of abundant -OH groups on the surface of electrode during the pre-activation process.

Then, VMSF was grown on p-GCEs by electrochemically assisted self-assembly (EASA). As a simple method to grow VMSF, EASA can quickly complete the growth of VMSF in a very short time (usually within 10 s). Due to the reaction of oxygen-containing functional groups (e.g., -OH) on p-GCE with silanol groups on VMSF, the film can be stably bound to the electrode surface. After VMSF growth, nanochannels are filled with surfactant micelles and the blocked electrode is obtained (denoted as SM@VMSF/p-GCE). When the SM is removed, VMSF/p-GCE with open nanochannel array is obtained.

**FIGURE 2:**
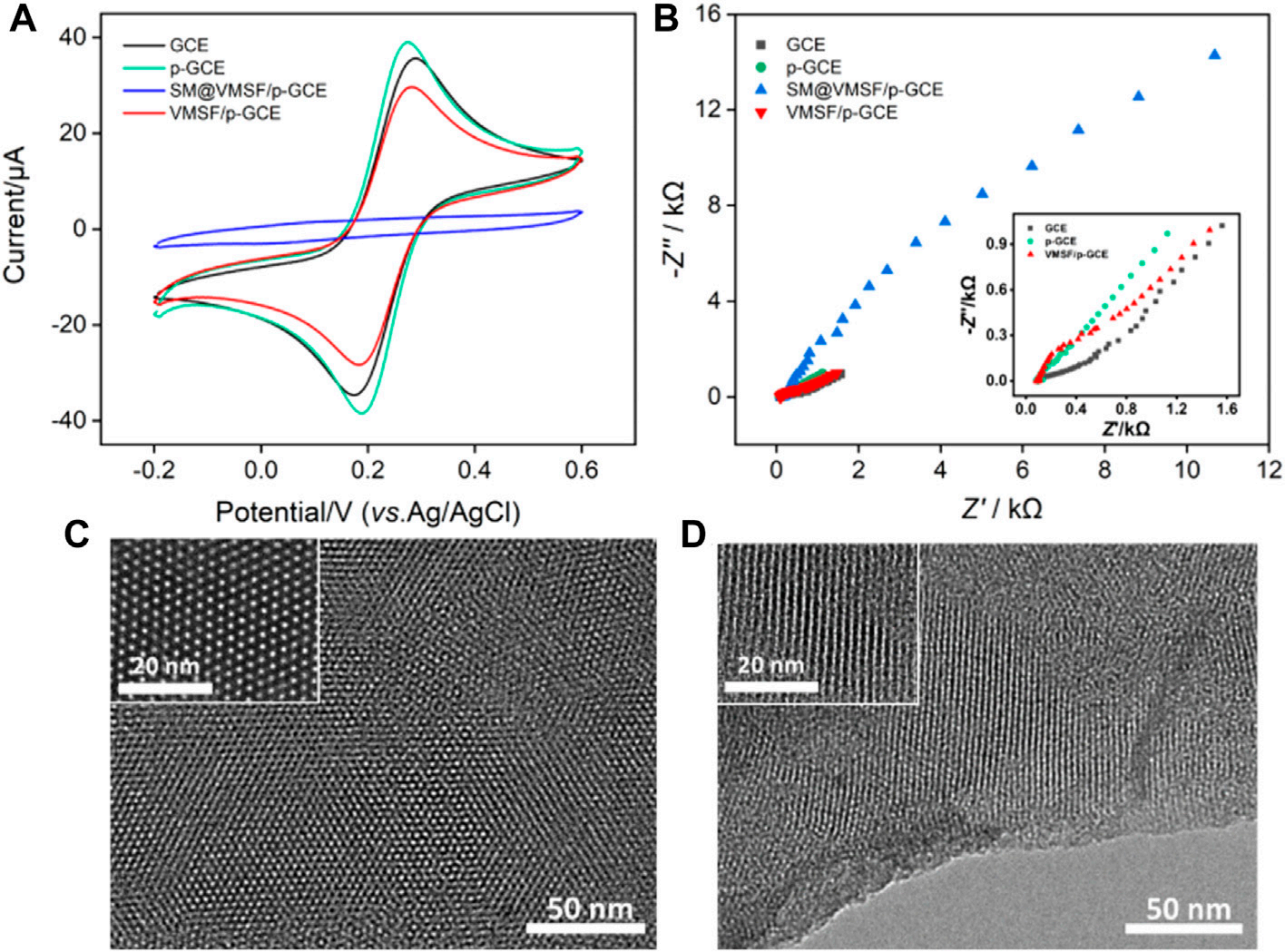
CV curves **(A)** and EIS plots **(B)** obtained on different electrodes in KCl (0.1 M) containing Fe(CN)_6_^3/4−^ (2.5 mM). Top-view **(C)** and cross-sectional **(D)** TEM images of VMSF at different magnification.

Cyclic voltammetry (CV) and electrochemical impedance spectroscopy (EIS) are used to investigate the changes of the electrode interface during the construction of the sensor. Figure 2A shows the CV curves of the standard redox probe (Fe(CN)_6_^3-/4-^) obtained on different electrodes. In comparison with bare GCE, p-GCE obtained after electrochemical pre-activation shows higher peak current and smaller peak-to-peak difference, indicating the improved electron transfer rate. After grown of VMSF, the Faradaic current signal of Fe(CN)_6_^3-/4-^ is hardly to be observed on SM@VMSF/p-GCE, indicating that the micelles block the nanochannel and prevent the diffusion of redox probes. This also proves that VMSF film covers the surface of p-GCE with integrity. After the removal of micelles, the electrochemical signal is restored on VMSF/p-GCE. Compared with p-GCE, VMSF/p-GCE exhibits slightly lower peak current because of the electrostatic repulsion between Fe(CN)_6_^3-/4-^ and negatively charged nanochannels of VMSF.

Figure 2B shows Nyquist plots recorded on different electrodes in Fe(CN)_6_^3-/4-^ solution. As shown, different electrodes have different electron transfer resistance (*R*_et_), which is related to the semicircle diameter of each curve. Since p-GCE has a higher electron transfer rate and hydrophilicity than GCE, the *R*_et_ obtained on p-GCE is smaller. In case of SM@VMSF/p-GCE, the *R*_et_ is extremely high because micelles block the nanochannels and prevent the diffuse of the probes to the surface of the electrode. On the contrary, VMSF/p-GCE, that has open nanochannel array exhibits slightly larger than that of p-GCE, proving the permeability of VMSF and the electrostatic repulsion effect towards negatively charged Fe(CN)_6_^3-/4-^. These results deduced from EIS measurement validate the conclusions obtained from CV experiments.

The morphology of the grown VMSF is investigated using transmission electron microscopy (TEM). As shown in Figure 2C, the top-view TEM images at different magnifications reveal uniformly distributed pores with hexagonal structure. The diameter of the nanopore is 2–3 nm. From the cross-sectional TEM image, it can be seen that the VMSF has an array of mesoporous nanochannels perpendicular to the substrate electrode (Figure 2D). These results demonstrate the successful growth of VMSF on p-GCE.

### 3.2 Electrochemical behavior of p-nitrophenol on vertically-ordered mesoporous silica-nanochannel film/p-glassy carbon electrode

**FIGURE 3:**
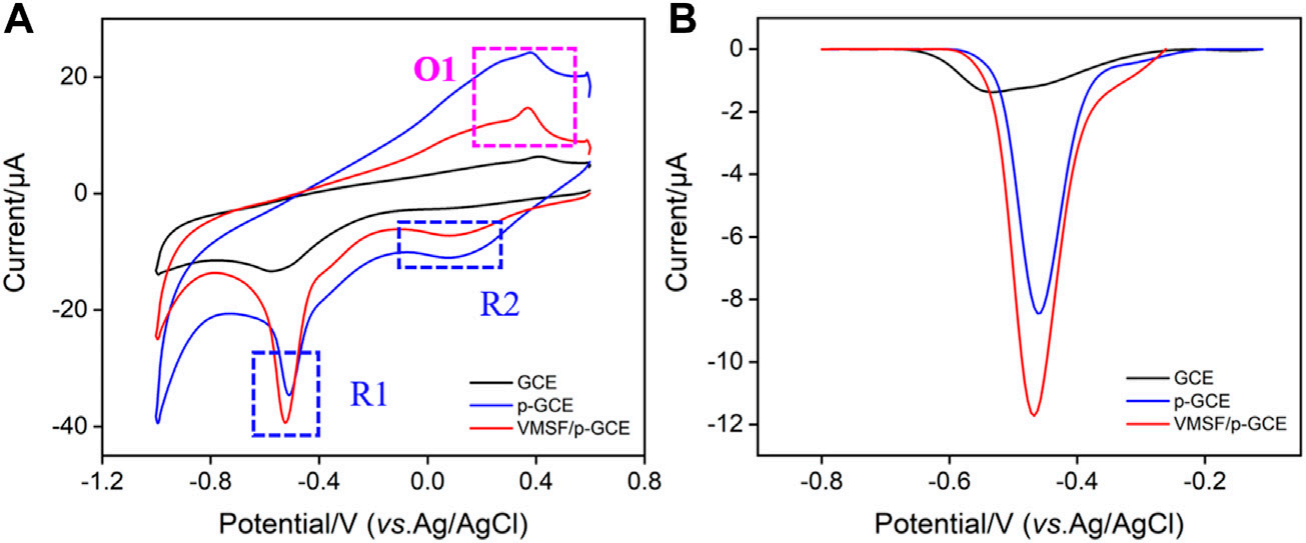
CV **(A)** and DPV **(B)** curves obtained on different electrodes in PBS (0.1 M, pH = 3) containing p-NP (30 μM).

The electrochemical behaviors of p-NP are investigated by CV and differential pulse voltammetry (DPV). Figure 3A shows the CV and DPV curves obtained on different electrodes in p-NP solution. As shown, the electrochemical process of p-NP on the electrode includes a pair of reversible redox processes (O1 and R2) and an irreversible reduction process (R1). Briefly, nitrophenol is reduced to hydroxylaminophenol (R1). With the change of potential, the coupled redox peak indicates that the reversible conversion between hydroxylaminophenol and nitrosophenol has occurred (R2 and O1) (Moraes et al., 2009; Zhang et al., 2018; Xuan et al., 2021). Bare GCE shows the lowest peak currents, while the electrochemical signal on p-GCE increases significantly. This is attributed to the increased active sites and electroactive area created in electrochemical pre-activation. The peak current of p-NP was further increased when VMSF was grown on p-GCE. In the tested acidic medium (pH = 3), dissociation of silanol groups (p*K*_a_ 2–3) is not significant. The enrichment of p-NP might be achieved through the hydrogen bonding interaction between the silanol groups on the pore walls and p-NP molecules (Singh et al., 2014; Yan et al., 2021), resulting in an enhanced electrochemical signal. Since the reduction peak current is largest near −0.46 V, the corresponding potential region is selected for DPV scanning. As shown in Figure 3B, the DPV curves demonstrate the consistent results.

**FIGURE 4:**
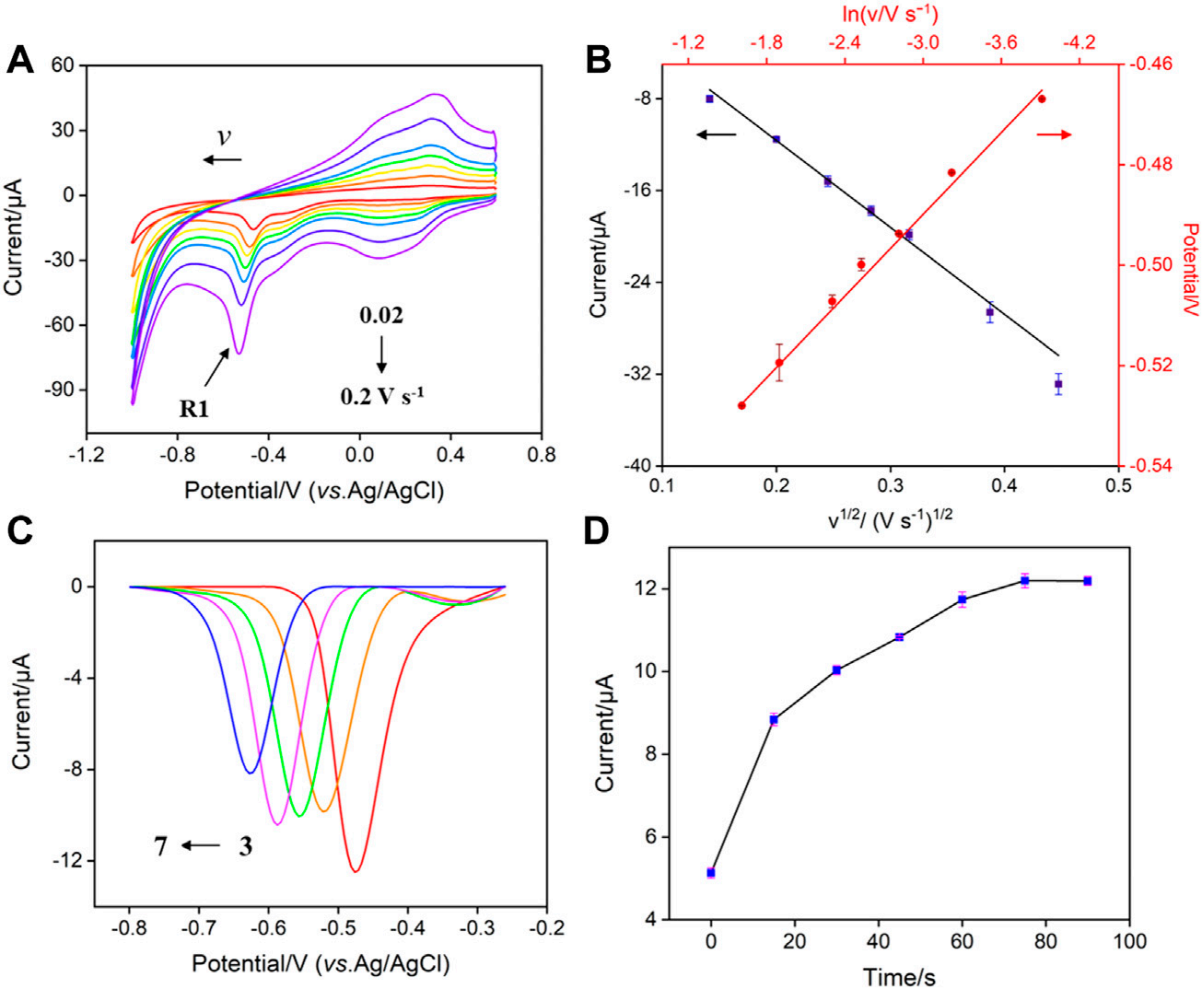
**(A)** CV curves obtained on VMSF/p-GCE at different scan rates (0.02–0.2 V s^−1^) in PBS (0.1 M, pH = 3) containing p-NP (30 μM) **(B)** Plots of *I*_R1_ vs*. ν* ^1/2^ and *E*_R1_ vs*.* ln *ν*. **(C)** DPV curves obtained on VMSF/p-GCE in p-NP (30 μM) solution with different pH. **(D)** The dependance of the peak current of p-NP (30 μM) on the enrichment time.

Figure 4A shows the CV curves of p-NP (30 μM) obtained on VMSF/p-GCE at different scan rates. The peak current (*I*) increases with increasing the scan rate (*v*). Taking R1 peak as a demonstration, the peak potential (*E*) shifts negatively as the scan rate increases. The peak current is linear with *v* ^1/2^ (Figure 4B, *I* = −75.7 *v* ^1/2^–3.49, *R* ^2^ = 0.997), demonstrating a diffusion-controlled electrochemical process. CV curves of low concentration of p-NP (0.1 μM) at different scan rates are also investigated. When the oxidation peak (O1) with high peak current is chosen, the same diffusion-controlled electrochemical process is also revealed (*I* = 89.6 *v* ^1/2^–14.8, *R* ^2^ = 0.996, Supplementary Figure S3) when the current was used. The slope is higher than that obtained at high concentration might indicate the different adsorption/diffusion ratio at different concentrations of analytes. In addition, the peak potential in Figure 4A can positively correlate with ln*v* through the linear equation (*E* = −0.0271 ln*v* −0.571, *R* ^2^ = 0.997). The number of electrons transferred in the irreversible reaction can be determined using Nicholson’s model Eqs. 1 and 2 (Cheng et al., 2021; Li et al., 2021).

$$E=E^{\theta }{-\text{M}}_{1}\left[\text{0}\text{.}78+\text{ln}\left(D^{1/2}{Ks}^{-1}\right)-\text{0}{\text{.}5\text{lnM}}_{1}\right]-\text{0}{\text{.}5\text{M}}_{1}Inv$$ (1)

M1=RT/[(1-α)nF] (2)

Where *E* ^θ^ reflects the formal potential; *D* is the diffusion coefficient; *Ks* corresponds to the electron transfer rate constant; *R* is the gas constant; *T* is the Kelvin temperature; *F* is the Faradayconstant; *n* and *α* are the number of transferred electrons and the electron transfer coefficient, respectively. Thus, the number of electrons transferred during this irreversible electroreduction is calculated to be 4, indicating that the electrochemical reduction of p-NP at the electrode involves the reaction of 4 electrons and 4 protons. The hydroxyl group on the nitrophenol is reduced to hydroxy-p-aminophenol. The corresponding electrochemical redox processes are illustrated below.

### 3.3 Optimization of the detection conditions

In order to obtain the best sensitivity for p-NP detection, the effect of pH value of the electrolyte solution on the detection of p-NP is investigated. Figure 4C shows the DPV curves of p-NP on VMSG/p-GCE at different pH. The peak current of p-NP is maximum at pH 3. This may be attributed to the strongest hydrogen bonding interaction between the silanol groups in nanochannels and p-NP. Thus, PBS (0.1 M, pH 3) was selected as the supporting electrolyte for further investigation. The effect of the enrichment time on the electrochemical signal of p-NP was also studied. As revealed in Figure 4D, the peak current of p-NP (30 μM) on VMSF/p-GCE has a plateau when the enrichment time is not less than 75 s. At lower p-NP concentration (0.1 μM), the adsorption equilibrium time is extended to 125 s (Supplementary Figure S2), indicating different adsorption-diffusion ratios at different p-NP concentrations. For fast detection, the enrichment time is set to 75 s.

### 3.4 Electrochemical detection of p-nitrophenol using vertically-ordered mesoporous silica-nanochannel film/p-glassy carbon electrode sensor

**FIGURE 5:**
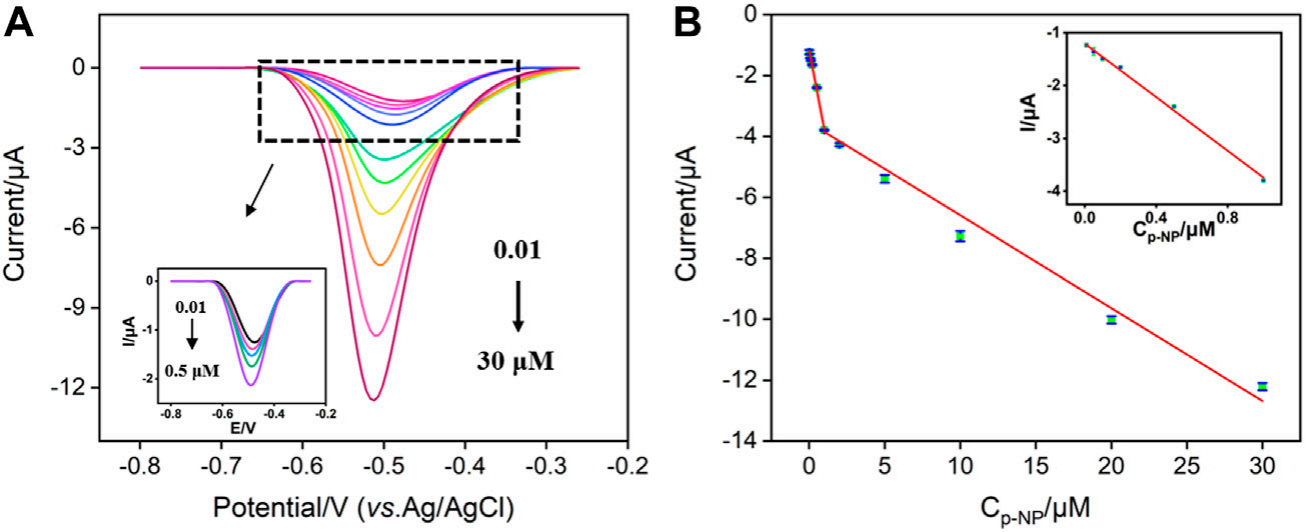
**(A)** DPV curves obtained on VMSF/p-GCE with different concentrations of p-NP. Inset is the magnified view of the DPV curves in the low-concentration region. **(B)** The calibration curve for the detection of p-NP. Inset is the calibration line at low concentration.

Under the optimal conditions, the constructed VMSF/p-GCE sensor is employed for electrochemical detection of p-NP. Figure 5A shows the DPV curves obtained on VMSF/p-GCE in presence of different concentrations of p-NP. When the concentration of p-NP is in the range of 10 nM–1 μM and 1–30 μM, the peak current (*I*) shows a good linear relationship with the concentration of p-NP (C_p-NP_) (Figure 5B, *I* = −2.59C_p-NP_ −1.17, *R* ^2^ = 0.996 and I = −0.305C_p-NP_ −3.54, *R* ^2^ = 0.992). As seen, the sensitivity decreases at high concentration range. Such phenomenon is commonly observed in many sensors attributable to different adsorption/diffusion ratio at high concentration of analyte resulting from the decreased active binding sites and mass transfer. The limit of detection (LOD) calculated using signal-to-noise ratio of three (S/N = 3) is 9.4 nM. Supplementary Table S1 lists the performance of different electrochemical sensors for the detection of p-NP. The LOD is lower than that obtained from copper nanoparticles and 4,4′-bpy, 4,4′-bipyridine modified gold electrode (CuNPs/4,4′-bpy/AuE) (Liu et al., 2017), nanostructure molecularly imprinted polyaniline modified graphene oxide composite (MIP-PANI/GO) (Saadati et al., 2018), polyvinylpyrrolidone@few-layer black phosphorus nanosheets modified GCE (PVP@BPNS/GCE) (Shen et al., 2021), selenide iron modified GCE (FeSe_2_/GCE) (Cheng et al., 2021), and delaminated titanium carbide/graphene modified GCE (D-Ti_3_C_2_T_X_/GR/GCE) (Wang et al., 2022b), but higher than that obtained on poly (arginine)/electrochemically *in situ* synthesized graphene modified screen printed electrode (*p*(Arg)/eG/SPE) (Li et al., 2015). In addition, the sensitivity and the preparation of the sensor are also compared. The developed VMSF/p-GCE sensor possessed advantages of high sensitivity and convenient preparation.

### 3.5 The antifouling ability, selectivity, and regeneration of vertically-ordered mesoporous silica-nanochannel film/p-glassy carbon electrode sensor

**FIGURE 6:**
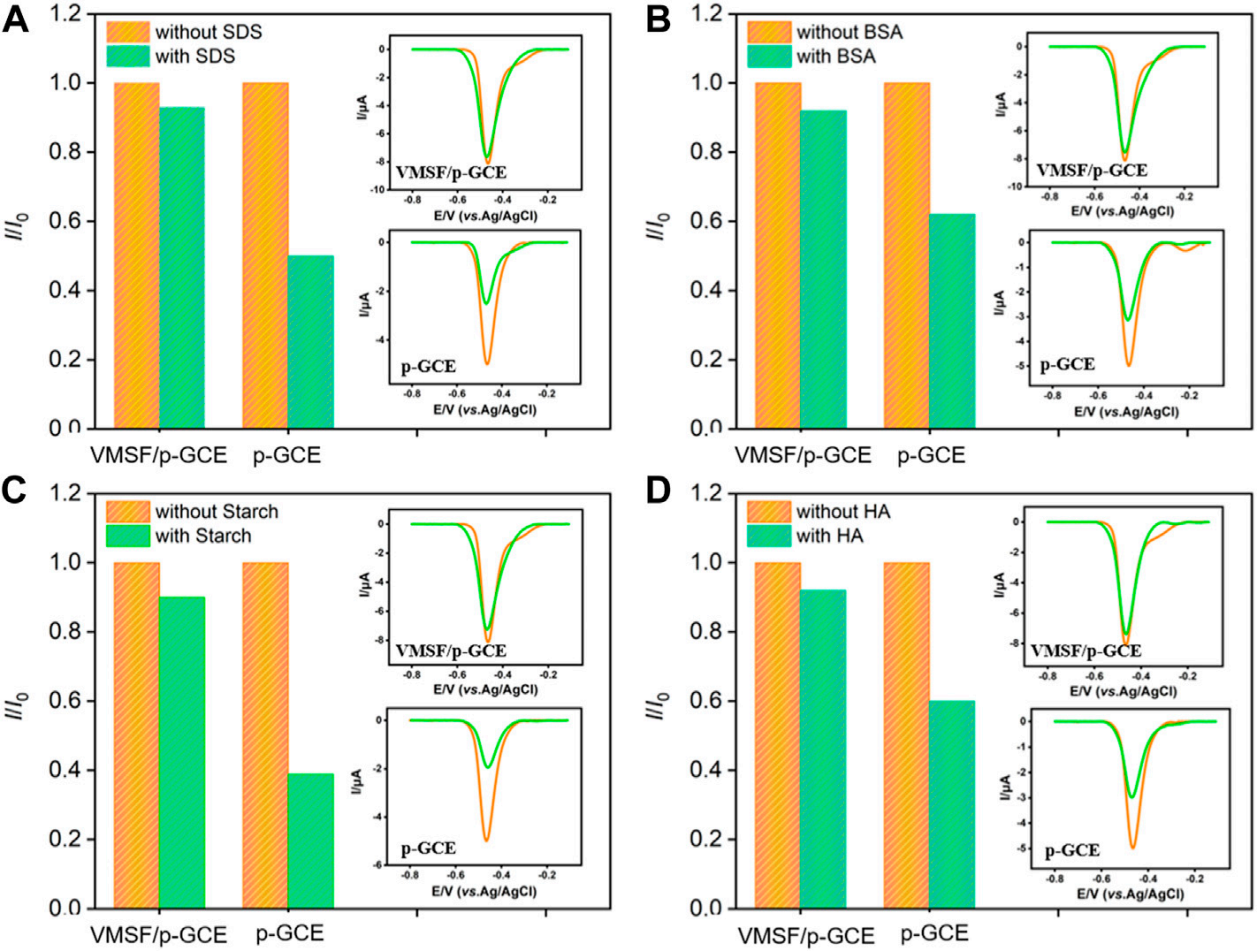
Normalized peak current ratio on VMSF/p-GCE or p-GCE towards p-NP (10 μM). *I* and *I*_0_ represent the currents obtained in the present and absence of 50 μg/ml of SDS **(A)**, BSA **(B)**, Starch **(C)** or HA **(D)** in PBS (0.1 M, pH = 3). The insets are the corresponding DPV curves obtained on p-GCE or VMSF/p-GCE in the absence and presence of the fouling species.

The antifouling ability of the electrode is investigated by using some common co-existing substances in complex environmental samples. Surfactant (sodium dodecyl sulfate-SDS as model), protein (bovine serum albumin-BSA as model), polysaccharide (starch as model) and humic acid (HA) are selected as the possible interferences. The peak current of p-NP on p-GCE and VMSF/p-GCE are measured before (*I*_0_) and after (*I*) addition of one of the above substances. As shown in Figure 6, only 50–70% of the initial peak current value is retained in the presence of these molecules, suggesting the electrode is heavily contaminated. This phenomenon leads to a significant decrease in the detection accuracy. On the contrary, the peak current obtained on VMSF/p-GCE in the presence of the possible interferences is very close to its initial signal, demonstrating a good antifouling ability of VMSF. This is attributed to the excellent size exclusion effect resulted from the highly uniform and ultrasmall nanopores of VMSF. Compared with p-GCE that has severe matrix effects in detection, VMSF/p-GCE exhibits great advantages in direct electroanalysis of complex samples.

**FIGURE 7:**
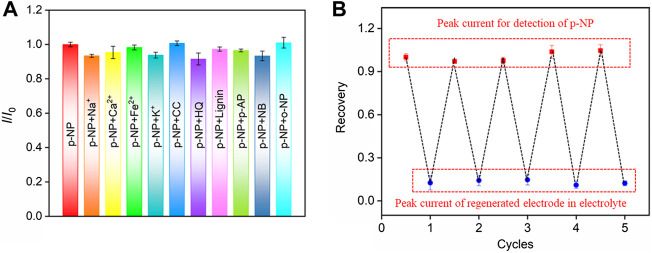
**(A)** The current ratio (*I*/*I*_0_) obtained on VMSF/p-GCE for detection of p-NP (20.0 μM) in the absence (*I*_0_) and presence (*I*) of the possible interfering species. The concentration of Na^+^, Ca^2+^, Fe^2+^, or K^+^ is 10-fold concentration of p-NP. The concentration of other added interfering species is 5-fold concentration of p-NP. **(B)** Regeneration of the VMSF/p-GCE. The first peak current is obtained using the original electrode towards p-NP. Other peak currents are obtained in electrolyte (bottom) or p-NP solution (top) using the regenerated electrodes. The concentration of p-NP is 20 μM.

The selectivity of the VMSF/p-GCE sensor for the detection of p-NP was further investigated. The effects of common metal ions (Na^+^, Ca^2+^, Fe^2+^, K^+^), the commonly environmental pollutants (catechol-CC, hydroquinone-HQ, lignin-Lignin, p-aminophenol-p-AP, nitrobenzene-NB), or an isomer of p-NP (ortho-nitrophenol, o-NP), on p-NP detection were studied. As shown in Figure 7A, even if some of the above substances are redox small molecules, they do not affect the detection of p-NP, indicating a good selectivity. This is attributable to the good potential resolution of the highly electroactive p-GCE.

The regeneration performance of the VMSF/p-GCE sensor towards p-NP detection was investigated. After p-NP assay, the electrode could be easily regenerated by stirring in HCl-ethanol solution for 5 min. Briefly, the signal value of p-NP (10 μM) detected by VMSF/p-GCE was recorded, and then the electrode was regenerated. The residual electrochemical signal on the regenerated electrode was measured in the supporting electrolyte. Afterwards, the electrode was used again for the measurement of p-NP. The detection and the following regeneration were performed for several cycles. As shown in Figure 7B, the peak current of p-NP obtained on the regenerated electrode is almost similar with that of the first detection. In addition, the relative standard deviation (RSD) of the peak current measured by the regenerated electrode for p-NP is 0.6%, indicating that VMSF/p-GCE sensor has excellent regeneration performance.

### 3.5 Real sample analysis

In order to verify the practical detection application of p-NP by the VMSF/p-GCE sensor, the detection of p-NP in workshop wasterwater is performed. The p-NP concentration values determined by the proposed electrochemical sensor and HPLC method were 7.20 ± 0.08 ng/ml (mean ± SD, *n* = 3) and 7.13 ± 0.05 ng/ml (mean ± SD, *n* = 3), respectively. The similar results indicates high reliability of the detection. In addition, the accuracy of p-NP detection in the pond water samples was evaluated by the spike recovery method (Supplementary Table S2). The recovery ranged from 95.8 to 106% with an RSD no more than 2.8%, indicating a high reliability in real sample analysis.

## 4 Conclusion

In summary, we have developed a simple electrochemical sensor based on integration vertically-ordered mesoporous silica-nanochannel films (VMSF) on electrochemical pre-activated GCE, which is able to achieve sensitive detection of p-nitrophenol (p-NP) with high selectivity and good anti-fouling ability. Electrochemical pre-activation of GCE is achieved by a simple and green electrochemical polarization. On the one hand, the pre-activation process increases the active area of the electrode and introduces active sites. On the other hand, it can realize stable binding of VMSF on the electrode surface. The electrochemical pre-activation process is simple and convenient to operate. VMSF is grown by electrochemically assisted self-assembly (EASA) method, which can complete film growth within 10 s. Thus, the strategy for the construction of VMSF/p-GCE sensor is simple and needs short preparation time. Due to the hydrogen bonding between VMSF and p-NP, VMSF nanochannel can enrich analytes, leading to significantly improved detection sensitivity. The size exclusion effect of ultra-small nanopores endows the VMSF/p-GCE sensor with excellent antifouling performance. The sensing electrode can also be rapidly and easily regenerated. This simple VMSF-based sensor might facilitate the facile fabrication of electrochemical sensing platform with high sensitivity, good antifouling, and easy regeneration.

## Data Availability

The original contributions presented in the study are included in the article/Supplementary Material, further inquiries can be directed to the corresponding authors.
