# Supplementary material for: Sensitive electrochemical detection of p-nitrophenol by pre-activated glassy carbon electrode integrated with silica nanochannel array film
Source: Front Chem. 2022 Aug 5;10:954748. doi: 10.3389/fchem.2022.954748 (PMC9388733; doi:10.3389/fchem.2022.954748)
Supplement: Supplementary file 1 [file DataSheet1.PDF]

TABLES

Table S1 Comparison between determination of p-NP using different electrode.

Electrode materials	*Method*	*Detection range* (μM)	*Sensitivity* (μA μM^{-1})	*Preparation time* (min)	*LOD* (nM)	*Ref.*
CuNPs/4,4'-bpy/AuE	I-T	1-500	7.413	1680	340	44
MIP-PANI/GO	CV	60-140	-	~20	2×10^4	45
PVP@BPNS/GCE	DPV	0.10–5.0	0.5107	260	28	46
$FeSe_2$/GCE	DPV	1-500	0.397	870	39.7	1
D-$Ti_3C_2T_X$/GR/GCE	DPV	1-175	0.2302	> 1440	169	47
P(Arg)/eG/SPE	I-T	5-200	0.032	~14	3.9	48
VMSF/p-GCE	DPV	0.01-1, 1-30	2.59	~10	9.5	This work

P(Arg), poly(arginine); eG, electrochemically *in situ* synthesized graphene; SPE, screen printed electrode; I-T, Amperometry; CuNPs, copper nanoparticles; 4,4'-bpy, 4,4′-bipyridine; AuE: gold electrode; MIP, imprinted polymers; PANI, polyaniline; GO, graphene oxide; CV: cyclic voltammetry; PVP, polyvinylpyrrolidone; BPNS, few-layer black phosphorus nanosheets; DPV: differential pulse voltammetry**;** $FeSe_2$, selenide iron; D-$Ti_3C_2T_X$, delaminated titanium carbide; GR; graphene; GCE; glassy carbon electrode.

Table S2 Determination of p-NP in pond water samples.

Sample	Spiked (μM)	Found (μM)	RSD (%, n=3)	Recovery (%)
pond water	0.0500	0.0479	2.6	95.8
	0.100	0.106	1.4	106
	1.00	1.03	2.8	103

FIGURES

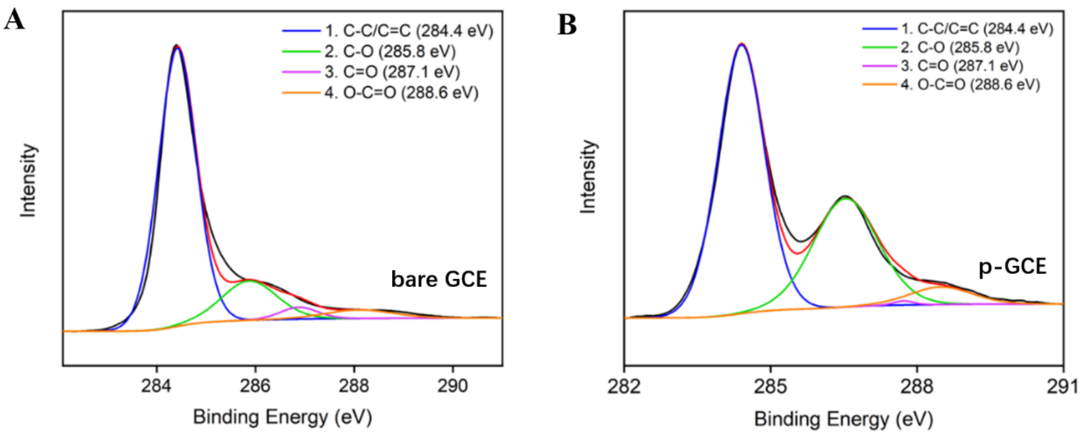

Figure S1 High-resolution C1s XPS spectra of bare GCE (A) and p-GCE (B).

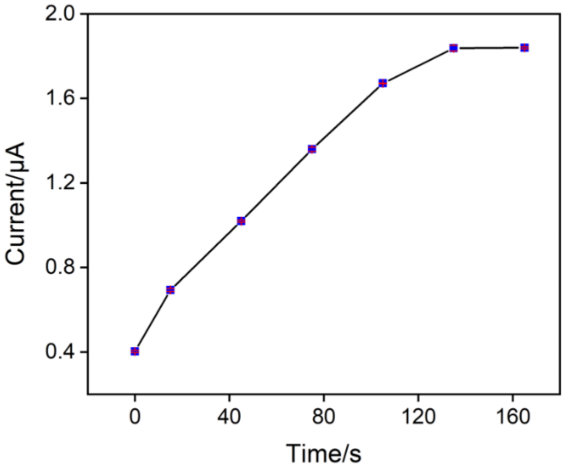

Figure S2 The dependance of the peak current of p-NP (30 μM) on the enrichment time.

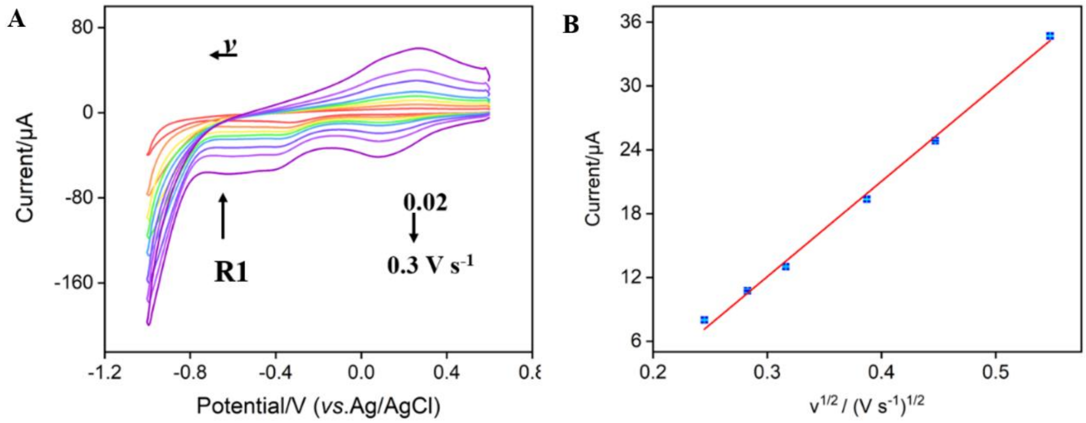

Figure S3 (A) CV curves obtained on VMSF/p-GCE at different scan rates (0.06 - 0.3 V s^{-1}) in PBS (0.1 M, pH=3) containing p-NP (0.1 μM). (B) Plots of I_{O1} *vs* $v^{1/2}$.
